# Supplementary material for: Divalent cations bind to phosphoinositides to induce ion and isomer specific propensities for nano-cluster initiation in bilayer membranes
Source: R Soc Open Sci. 2020 May 20;7(5):192208. doi: 10.1098/rsos.192208 (PMC7277276; doi:10.1098/rsos.192208)
Supplement: Supplementary Information [file rsos192208supp1.pdf]

Divalent cations bind to phosphoinositides to induce ion and isomer specific propensities for nano-cluster initiation in bilayer membranes

Ryan P. Bradley[★,1], David R. Slochower[★,2], Paul A. Janmey[3], and Ravi Radhakrishnan[1,4,†]

[★]These authors contributed equally
[1]Department of Chemical and Biomolecular Engineering, University of Pennsylvania, Philadelphia PA 19104
[2]Graduate Group in Biochemistry and Molecular Biophysics, University of Pennsylvania, Philadelphia PA 19104
[3]Department of Physiology, University of Pennsylvania, Philadelphia, PA 19104
[4]Department of Bioengineering, University of Pennsylvania, Philadelphia PA 19104
[†]Email: rradhak@seas.upenn.edu

Electronic Supplementary Information (ESI)

Supplementary Videos

SV1

See attached in ESI. Movie of a typical molecular dynamics simulation. The waters are not shown for clarity.

SV2

See attached in ESI. Movie comparing hydration shell dynamics of calcium (blue) and magnesium (pink) ions bound to PIP2 in a bilayer.

Supplementary Table

conditions			number of molecules																duration
PI species	cation	type	POPC	DOPE	DOPC	DOPS	Cholesterol	$PI(4,5)P_2$	$PI(3,5)P_2$	$PI(4,5)P_2^{-3}$	$PI(4,5)P_2^{-5}$	PI	K^+	Na^+	Mg^{2+}	Ca^{2+}	Cl^-	H_2O	time (ns)
$PI(4,5)P_2$	Na^+	sym	-	-	576	144	-	80	-	-	-	-	-	848	-	-	384	83440	100
$PI(4,5)P_2$	Mg^{2+}	sym	-	-	576	144	-	80	-	-	-	-	-	-	522	-	580	82338	100
$PI(4,5)P_2$	Ca^{2+}	sym	-	-	576	144	-	80	-	-	-	-	-	-	-	522	580	82338	100
$PI(4,5)P_2$	Mg^{2+}	AC	300	200	-	60	200	40	-	-	-	-	-	-	308	-	396	59445	100 (×2)<br>500
$PI(4,5)P_2$	Ca^{2+}	AC	300	200	-	60	200	40	-	-	-	-	-	-	-	308	396	59445	100 (×2)<br>500
$PI(3,5)P_2$	Mg^{2+}	AC	300	200	-	60	200	-	40	-	-	-	-	-	308	-	396	59445	100
$PI(3,5)P_2$	Ca^{2+}	AC	300	200	-	60	200	-	40	-	-	-	-	-	-	308	396	59445	100
$PI(4,5)P_2$	K^+	AC	300	200	-	60	200	40	-	-	-	-	495	-	-	-	275	60149	100
PI	Ca^{2+}	AC	300	200	-	60	200	-	-	-	-	40	-	-	-	294	488	60277	100

Table S1: This table enumerates the composition for each simulation used in this study. The type refers to either the asymmetric compositions (AC) which include cholesterol in both leaflets and PIP_2 or PI in the inner leaflet, or the symmetric (sym) bilayers, with the same composition in both leaflets. Selected figures from the main text make direct comparisons between the longest, 500ns trajectories as well as separate comparisons between the additional 100ns trajectories where applicable.

Supplementary Figures

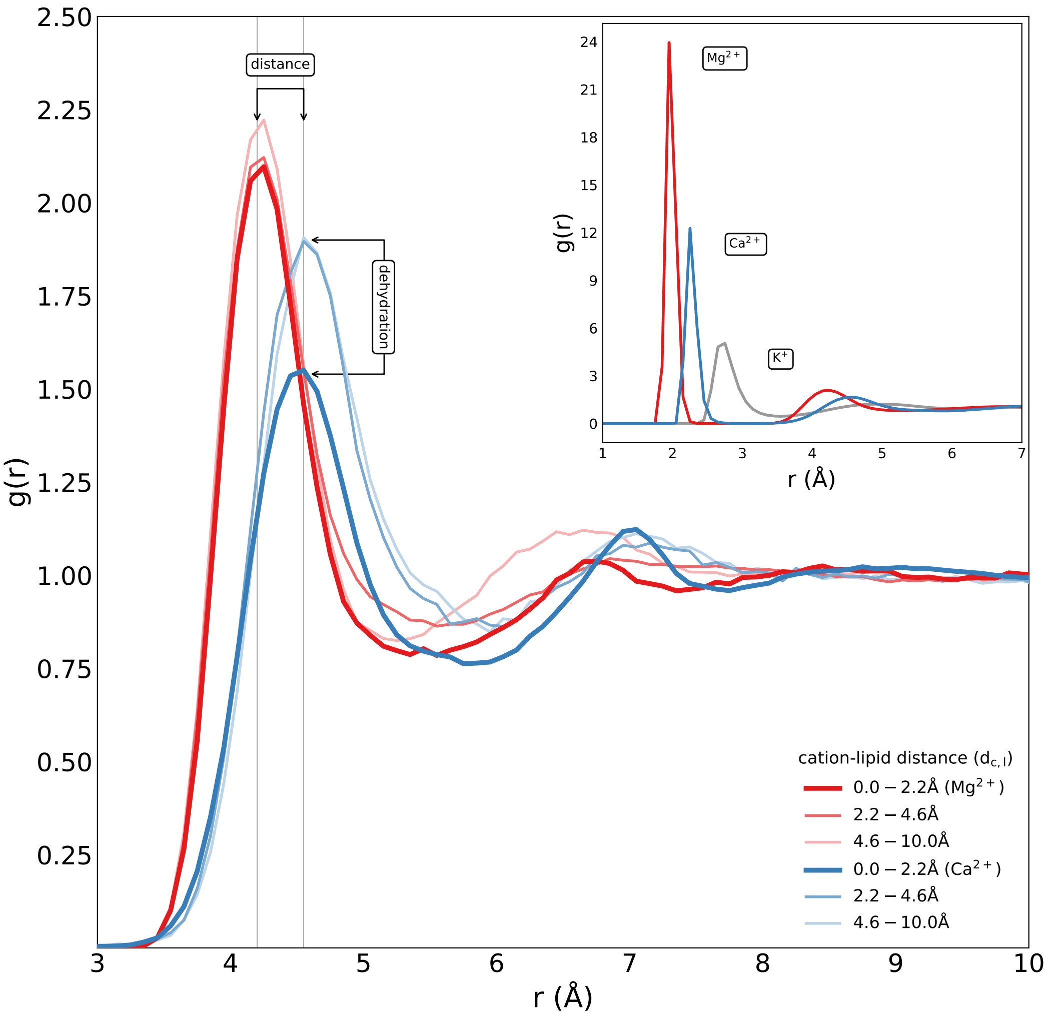

Figure S1: The radial distribution function between the ions and solvent. The inset shows both the first and second hydration shells for all ions, while the main plot shows slight differences in the second solvation shell according to the minimum ion-lipid distances (lighter curves are closer to lipids). Ca^{2+} shows more variation with lipid distances, suggesting a sharper dehydration transition. We also find that Ca^{2+} binds at a larger distance in both shells, and that it has less specific and numerous bonds with these waters, hence the shallower, broader peak.

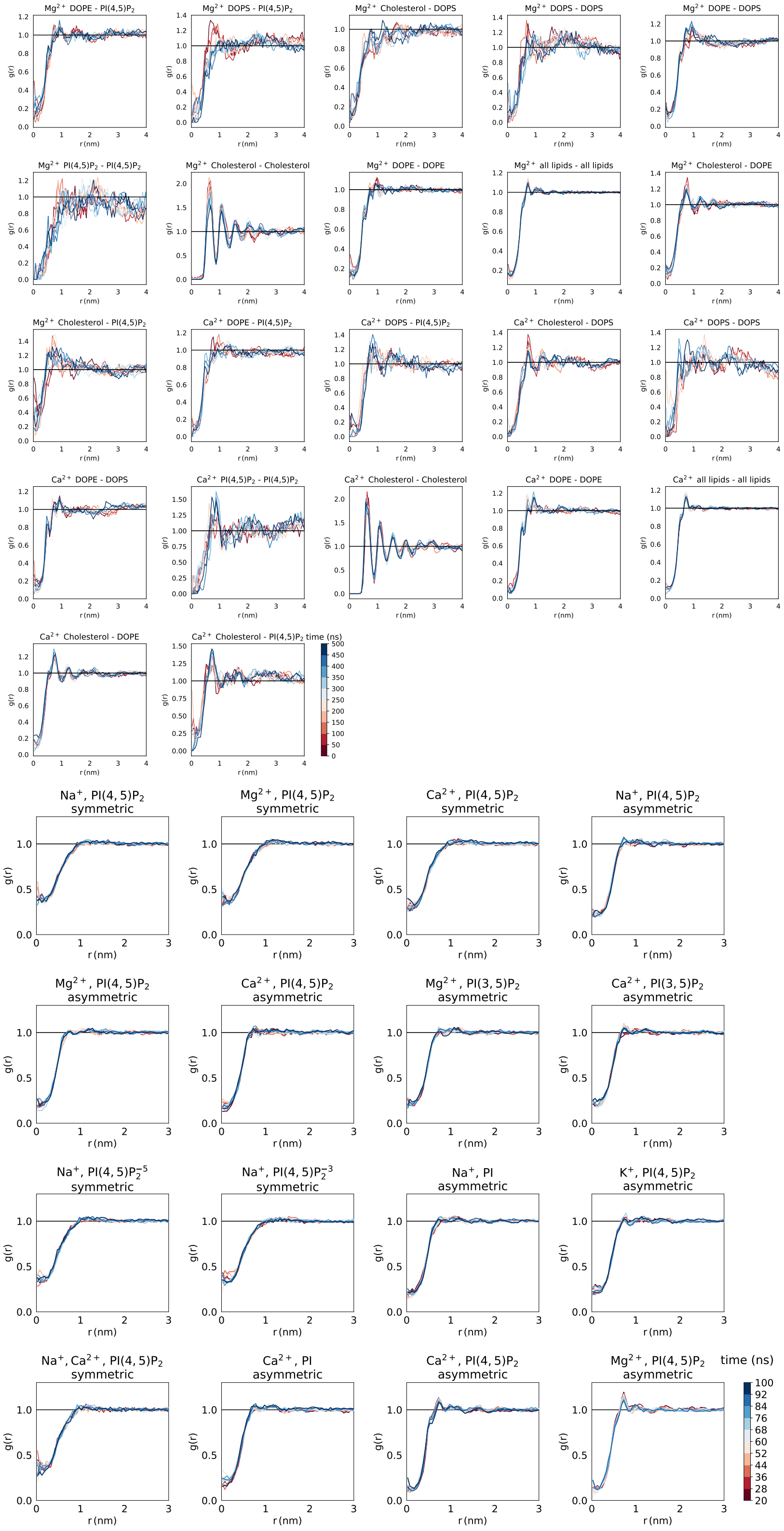

Figure S2: (top panels) The radial distribution functions computed for 50 ns length segments of the trajectories showing convergence is reached in the simulations after 200 ns. (bottom panels) For comparison, we also show plots for our shorter 100 ns simulations in 20 ns slices.

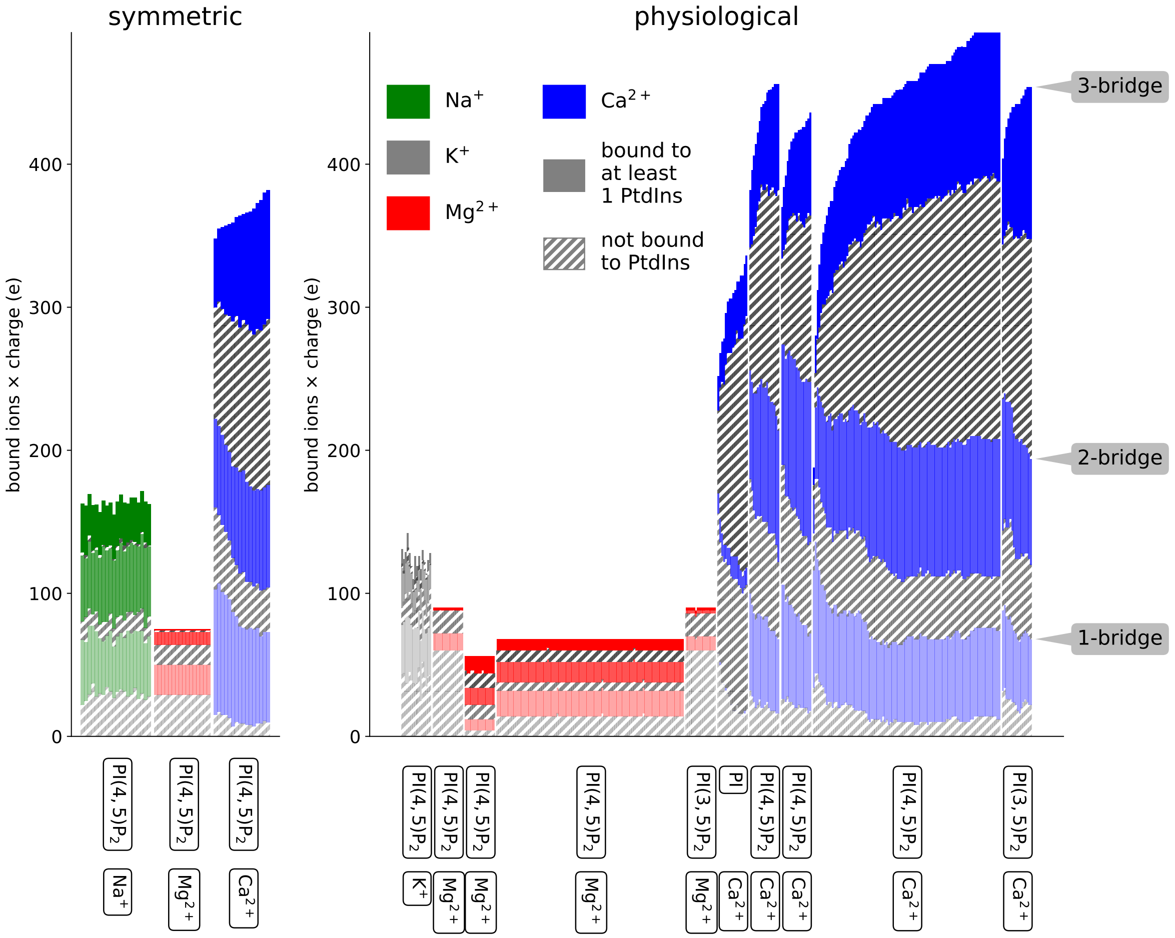

Figure S3: Charging curves for all simulations, including both symmetric (left) and asymmetric (right) bilayers. These counts are scaled by the total charge in order to emphasize the difference in charge density due to each ion. The two long-running (500 ns) simulations were presented in main text. For clarity, whave removed time from the horizontal axis, however each thin bar (a point in the time series) represents 5 ns. The longest simulations run for 500ns, while most of the shorter ones include 80 ns of sampling time (those that do not are noted in Table S1). We define bound ions according to the radius of the first solvation shell, which is 3.0 Å for Na^+ and K^+, 2.3 Å for Mg^{2+}, and 2.6 Å for Ca^{2+}. Solid, colored bars represent lipids which are bound to at least one PIP_2 species (labelled "Ptdins" because we have included PI), while gray, hatched bars represent cations bound to other lipids. We have excluded the outer leaflet in the asymmetric bilayers on the right panel. Note that the protonation state modulates the amount of bound Na^+ shown on the symmetric bilayers (left) consistent with the difference in charge. When comparing the product of bound ions and charge between symmetric and assymmetric bilayers, we find that the latter are roughly 19% higher when Ca^{2+} is present. This difference is attributed to either the presence of cholesterol, or the difference in leaflet composition asymmetry.

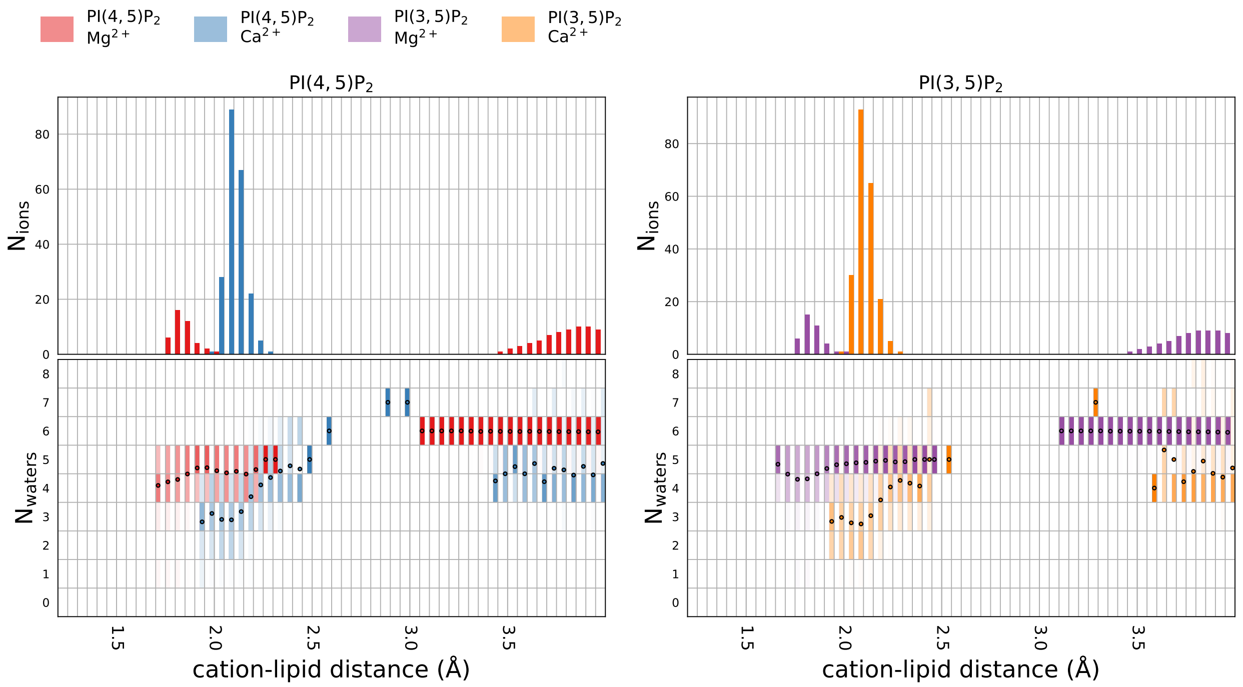

Figure S4: Comparison of ion-water solvation shells for Ca^{2+} and Mg^{2+} in the presence of $PI(4,5)P_2$ (left) and $PI(3,5)P_2$. We find very little difference in the structure of the solvation shell between PIP_2 isomers. This figure uses the same format as Figure on hydration from the main text.

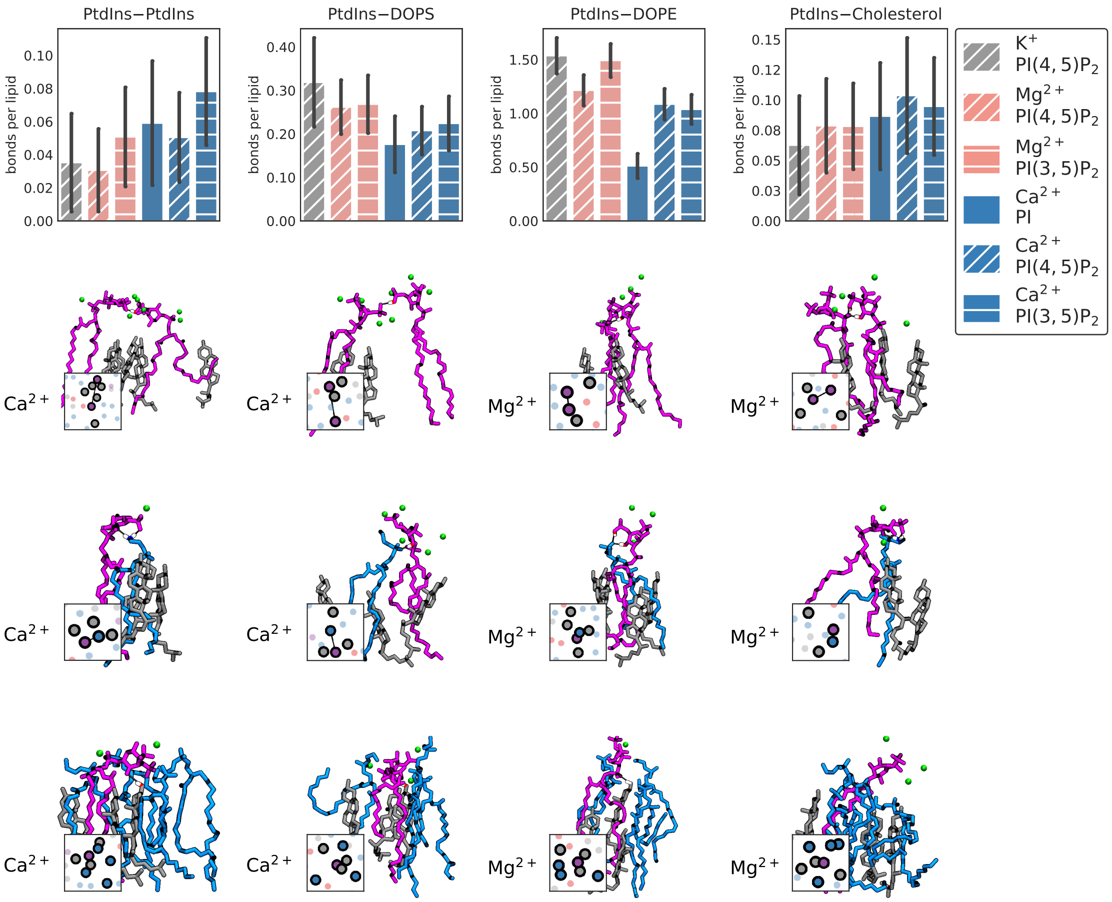

Figure S5: Hydrogen bonds between lipid species for different simulation conditions, reported as bonds per PIP_2 (top row). Snapshots of the most common hydrogen bonds formed between lipids in the presence of either Ca^{2+} or Mg^{2+} are shown beneath. The first row of snapshots shows $PIP_2 - PIP_2$ bonds with nearby cholesterol, the second row shows PIP_2 − DOPE bonds with nearby cholesterol, and the third row shows PIP_2 − Cholesterol bonds with nearby DOPE. We find that DOPE is the most likely hydrogen bond partner, however PIP_2 forms bonds with other PIP_2 and cholesterol, and this effect is accentuated slightly when Ca^{2+} is present. It is notable that while PI has fewer sites for hydrogen bonding, it forms more hydrogen bonds with other PI molecules than the other isoforms (see green bar, top left panel). The hydrogen bonding counts for systems not shown here are found in Figure S6.

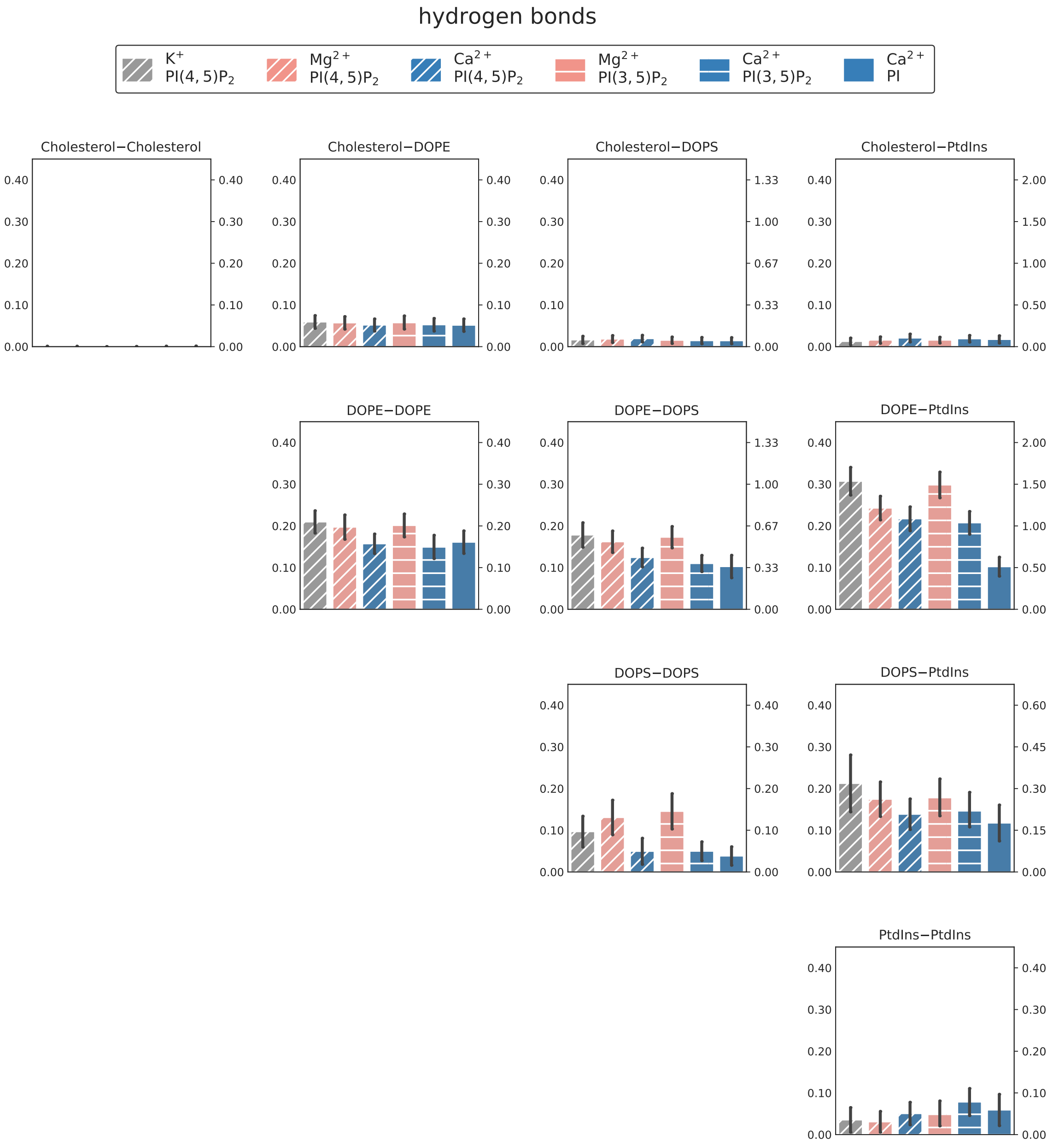

Figure S6: Hydrogen bond counts normalized by lipid. For each pair of lipids, the left axis tells you the average number of bonds for the first lipid in the pair, while the right axis provides the average number of bonds for the second lipid in the pair. For example, we find that each PIP_2 forms 1.5 bonds on average with DOPE when K^+ is present, according to the axis on the right. Alternately, each DOPE forms 0.3 bonds with PIP_2, according to the left axis.

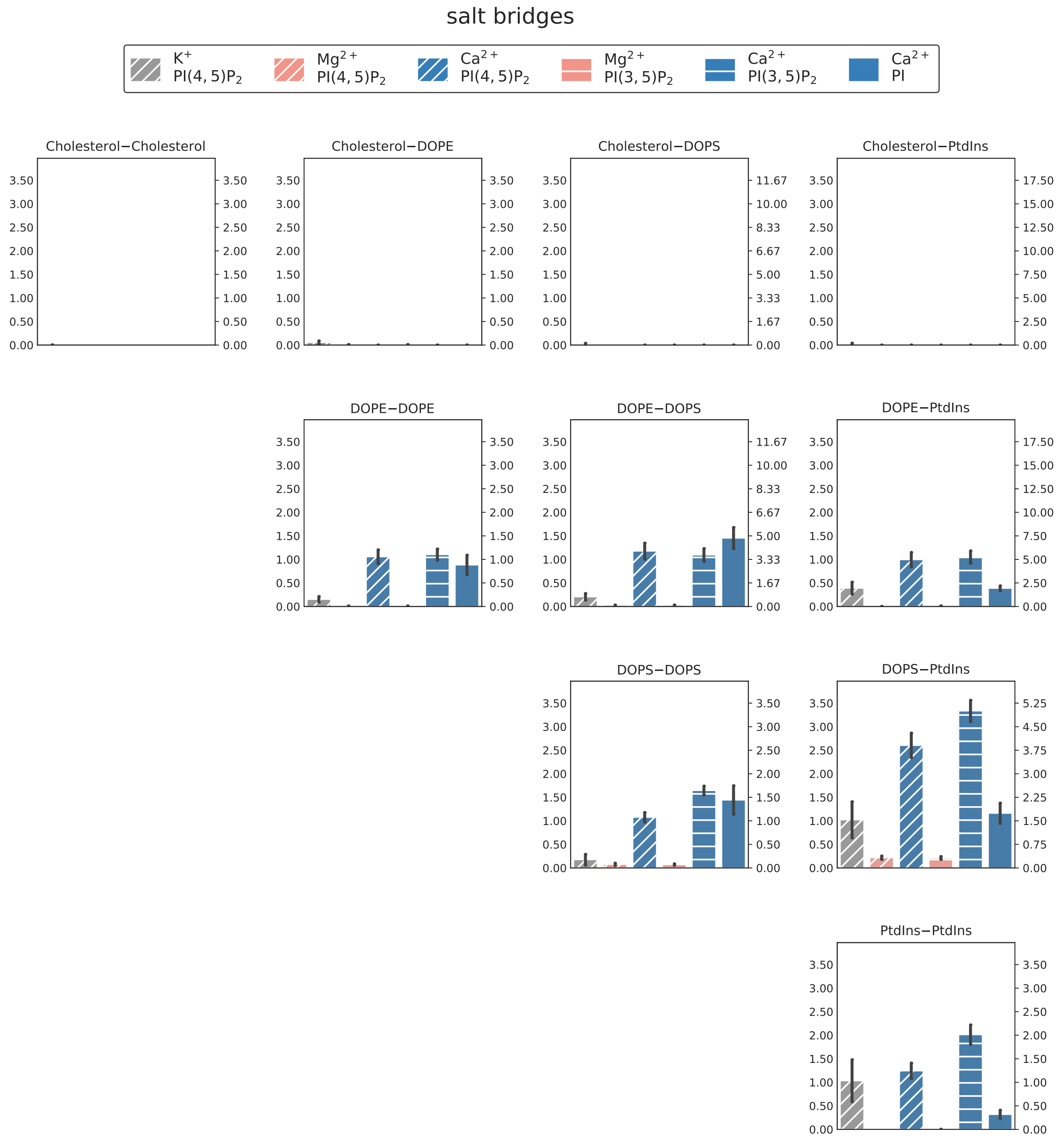

Figure S7: Normalized salt bridges measured with a cutoff of 3.4 Å. As with Figure S6, the left and right axes describe the number of bonds per lipid for the first and second lipid in the pair, respectively. We find that sodium and calcium form the most lipid-cation-lipid salt bridges.

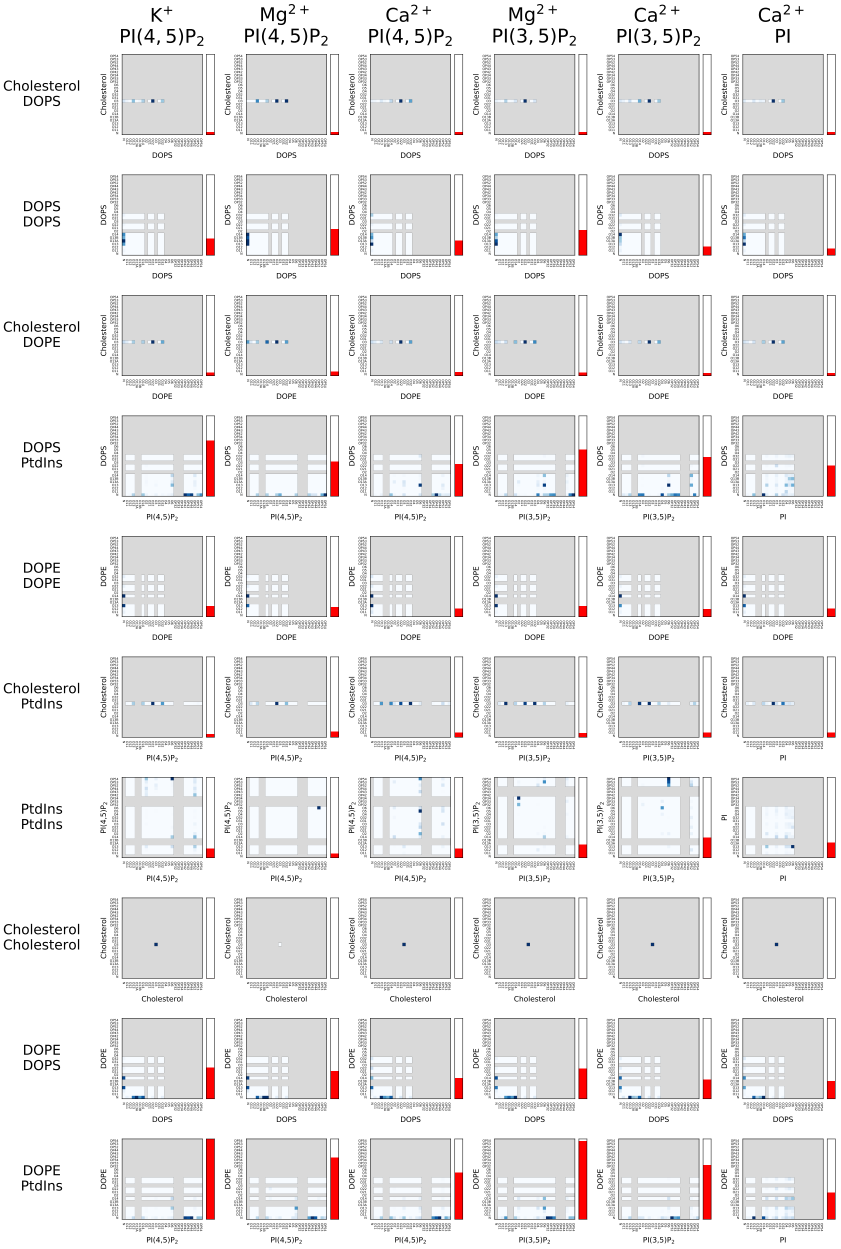

Figure S8: Atoms participating in lipid-lipid hydrogen bonds. Each tile shows a heat map of the number of hydrogen bonds for each atom type. The tiles are organized by lipid pairs (rows) and simulation composition (columns). Since there are different numbers of each lipid type, the red bars show a relative score (in bonds per donor per acceptor) that reflects the total propensity of this type of bond to form. These propensities match the bonds per lipid shown in Figure S6. Atoms which are absent from a particular lipid are masked in gray.

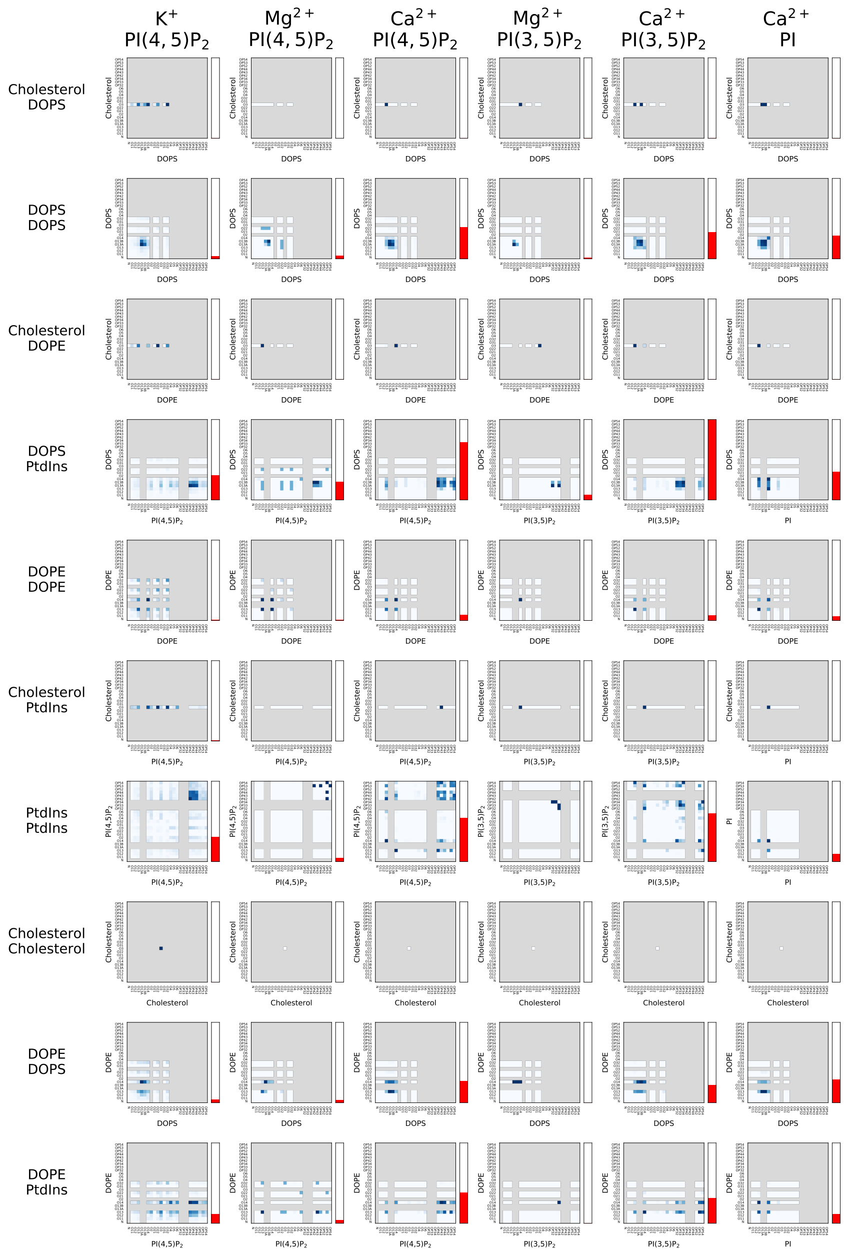

Figure S9: Atoms participating in lipid-cation-lipid salt bridges with a lipid-lipid cutoff of 3.4 Å. The tiles are organized by lipid pairs (rows) and simulation composition (columns). Since there are different numbers of each lipid type, the red bars show a relative score (in bonds per donor per acceptor) that reflects the total propensity of this type of bond to form. These propensities match the bonds per lipid shown in Figure S7. Atoms which are absent from a particular lipid are shown in gray.

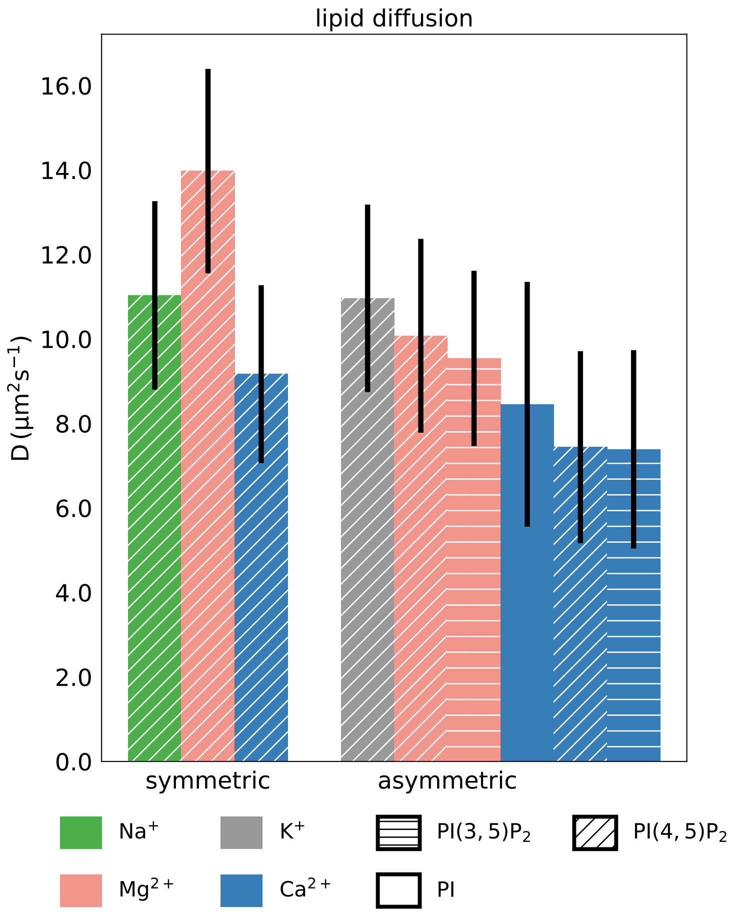

Figure S10: Lipid diffusion rates for both assymmetric and symmetric bilayers. Selected results are also plotted in the main text.

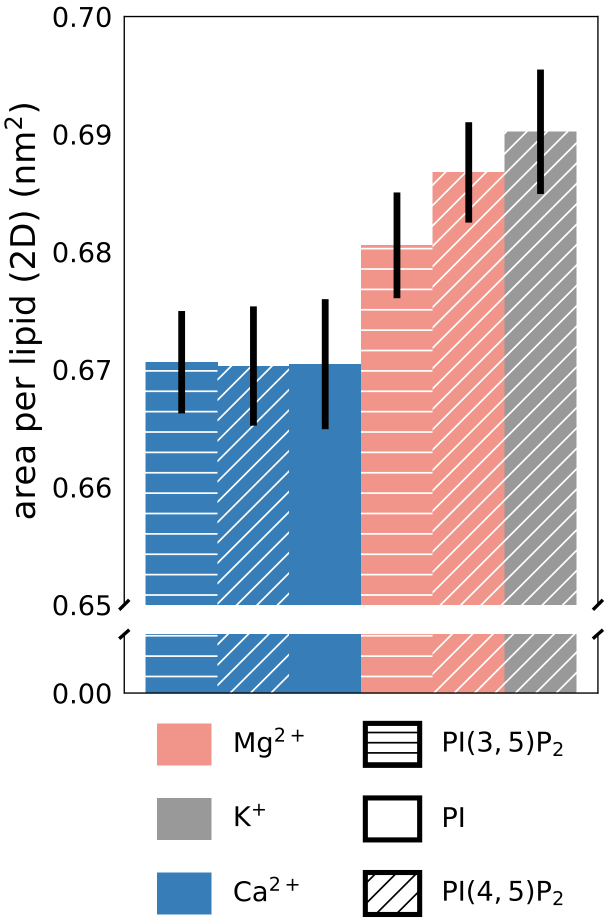

Figure S11: Projected, two-dimensional lipid areas for each simulation according to condition.

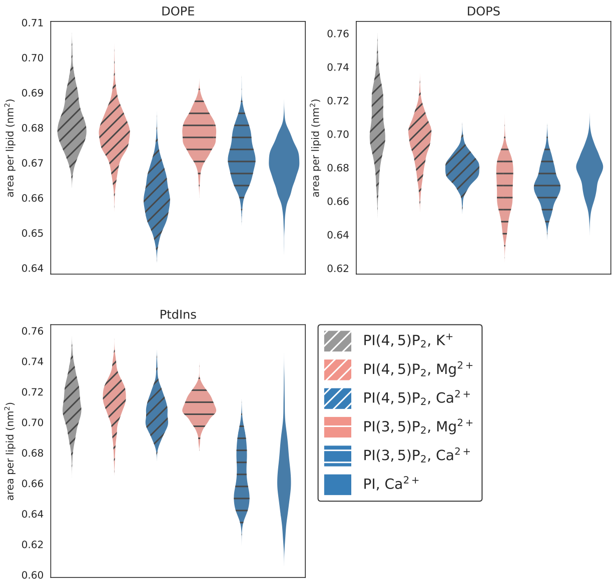

Figure S12: Lipid area distributions organized by lipid species. These distributions show the distribution of three-dimensional lipid areas across individual lipids. The results capture the extent to which the bulky PPI headgroups produce additional leaflet area and thereby bulge into the solvent more than the others. The averages across these three-dimensional area distributions provides the summary shown in the main text.

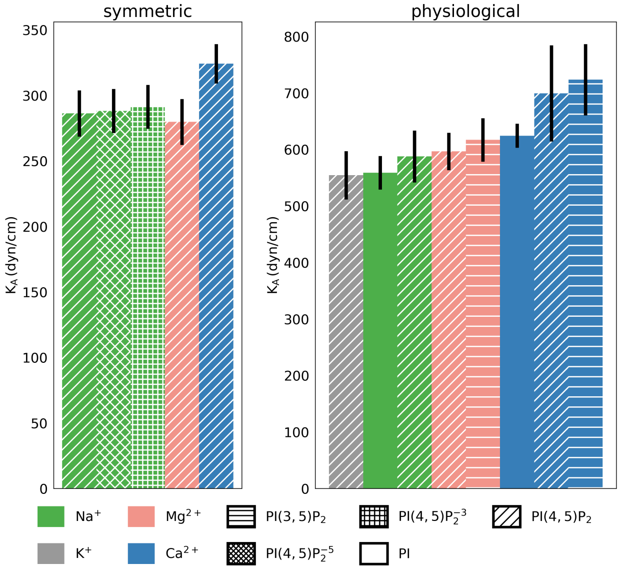

Figure S13: Area compressibility (K_A) for each simulation computed from per-lipid area fluctuations according to the formula $K_A = 2ak_BT\left(\sigma_a{}^2\right)^{-1}$, where a is the area per lipid, $\sigma_a{}^2$ is the variance in the area per lipid, and N is the total number of molecules in the bilayer. Lipid area fluctuations are computed from the centers of mass excluding the lipid head groups. Area compressibility is highest when cholesterol is present. We also find that Ca^{2+} increases the compressibility when PIP_2, but not PI, is present.

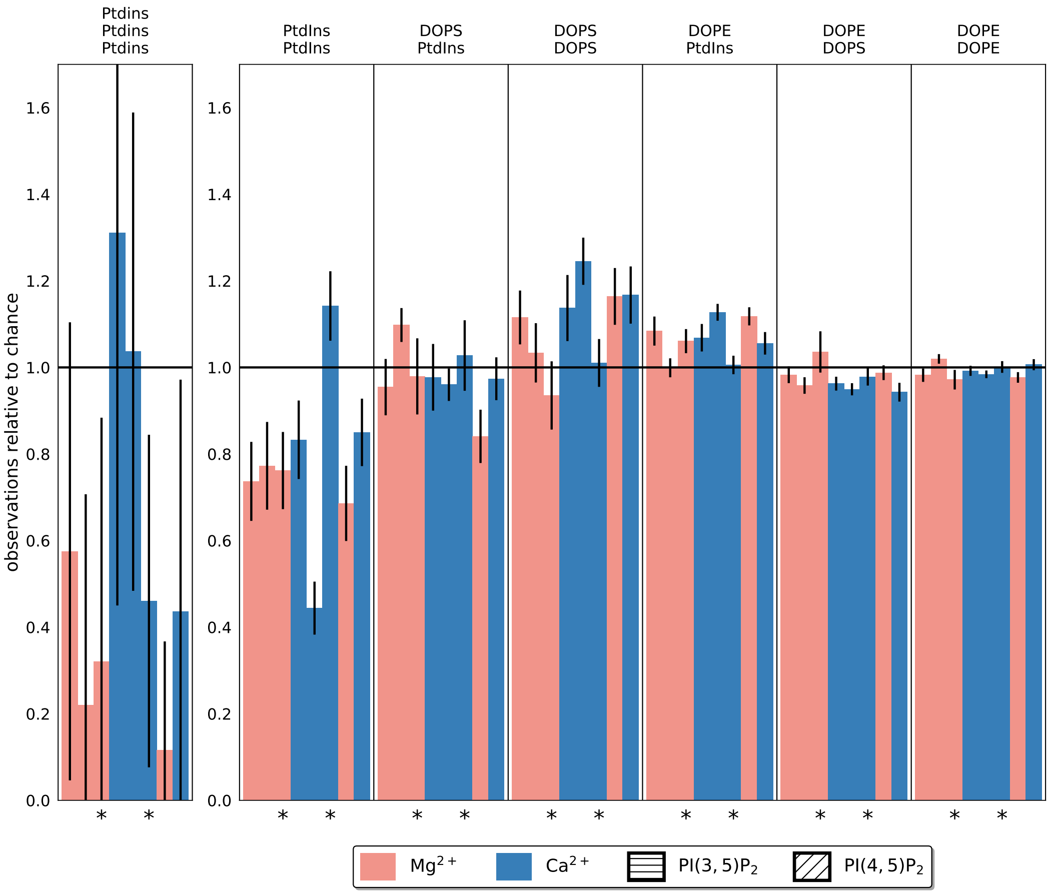

Figure S14: Observed numbers of triplets (left) or pairs (right) for various species in simulations of asymmetric bilayers. The lipid species participating in each association are noted above the plots. This plot includes two 100 ns replicates of both divalent cations along with $PI(4,5)P_2$, along with additional 500 ns trajectory noted with an asterisk ($*$) underneath, for each cation. We have also included two 100 ns replicates containing $PI(3,5)P_2$ (horizontal hatching). The score represents the proportion of observed associations relative to chance, where 1.0 represents the likelihood of seeing each pair or triplet in a randomized triangulation with the same composition. Scores above unity indicate a preference for that association, while scores below unity represent associations which are disfavored. Error bars represent the standard deviation of the number of each pair or triplet. This figure depicts all available pairs, while additional triplets from this dataset are not pictured.
